# (±)-*trans*-3-Oxo-1,2,3,4,4a,9,10,10a-octa­hydro­phenanthrene-10a-carboxylic acid: catemeric hydrogen bonding in a δ-keto acid

**DOI:** 10.1107/S1600536808026639

**Published:** 2008-08-23

**Authors:** Mark Davison, Roger A. Lalancette, Hugh W. Thompson, Alan J. Miller

**Affiliations:** a Carl A. Olson Memorial Laboratories, Department of Chemistry, Rutgers University, Newark, NJ 07102, USA

## Abstract

The title compound, C_15_H_16_O_3_, aggregates as hydrogen-bonded catemers progressing from each carboxyl to the ketone of a screw-related neighbor [O⋯O = 2.6675 (14) Å and O—H⋯O = 170°]. Two parallel centrosymmetrically related single-strand hydrogen-bonding helices proceed through the cell in the *b*-axis direction. The packing includes three inter­molecular C—H⋯O=C close contacts, involving both the ketone and the carboxyl group. The structure is isomorphous with that of the previously described Δ^4^ α,β-unsaturated ketone.

## Related literature

For related literature, see: Allen *et al.* (1999 ▶); Borthwick (1980 ▶); Gavezzotti & Filippini (1994 ▶); Leiserowitz (1976 ▶); Miller *et al.* (1999 ▶); Steiner (1997 ▶); Thompson & McPherson (1977 ▶); Thompson & Shah (1983 ▶).
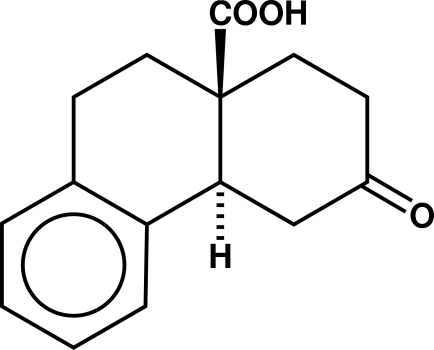


## Experimental

### 

#### Crystal data

C_15_H_16_O_3_
*M* *_r_* = 244.28
Monoclinic, 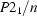

*a* = 9.7172 (4) Å
*b* = 12.2735 (6) Å
*c* = 10.4867 (5) Å
β = 102.6764 (19)°
*V* = 1220.20 (10) Å^3^
*Z* = 4
Cu *K*α radiation
μ = 0.74 mm^−1^
*T* = 100 (2) K
0.41 × 0.38 × 0.36 mm

#### Data collection

Bruker SMART CCD APEXII area-detector diffractometer
Absorption correction: multi-scan (*SADABS*; Sheldrick, 2001 ▶) *T*_min_ = 0.750, *T*_max_ = 0.775
7219 measured reflections
2106 independent reflections
2019 reflections with *I* > 2σ(*I*)
*R*_int_ = 0.019

#### Refinement

*R*[*F* ^2^ > 2σ(*F* ^2^)] = 0.039
*wR*(*F* ^2^) = 0.108
*S* = 1.05
2106 reflections
165 parameters
H-atom parameters constrained
Δρ_max_ = 0.25 e Å^−3^
Δρ_min_ = −0.20 e Å^−3^

### 

Data collection: *APEX2* (Bruker, 2006 ▶); cell refinement: *APEX2*; data reduction: *SAINT* (Bruker, 2005 ▶); program(s) used to solve structure: *SHELXTL* (Sheldrick, 2008 ▶); program(s) used to refine structure: *SHELXTL*; molecular graphics: *SHELXTL*; software used to prepare material for publication: *SHELXTL*.

## Supplementary Material

Crystal structure: contains datablocks I, global. DOI: 10.1107/S1600536808026639/fl2217sup1.cif

Structure factors: contains datablocks I. DOI: 10.1107/S1600536808026639/fl2217Isup2.hkl

Additional supplementary materials:  crystallographic information; 3D view; checkCIF report

## Figures and Tables

**Table 1:** Hydrogen-bond geometry (Å, °)

*D*—H⋯*A*	*D*—H	H⋯*A*	*D*⋯*A*	*D*—H⋯*A*
O3—H3⋯O1^i^	0.84	1.84	2.6675 (14)	170
C2—H2*A*⋯O2^ii^	0.99	2.45	3.3817 (16)	156
C4—H4*B*⋯O2^iii^	0.99	2.60	3.5273 (18)	156
C8—H8*A*⋯O1^iv^	0.95	2.55	3.2625 (17)	132

Symmetry codes: (i) 
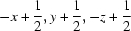
; (ii) 
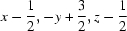
; (iii) 
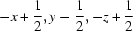
; (iv) 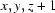.

**Fig. 1.**
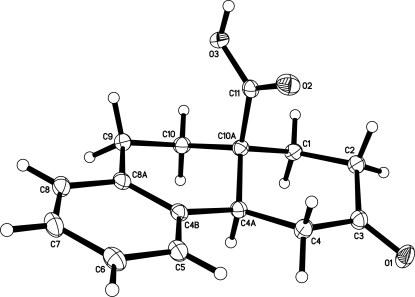
The asymmetric unit of (I), with its numbering. Displacement ellipsoids are drawn at the 30% probability level.

**Fig. 2.**
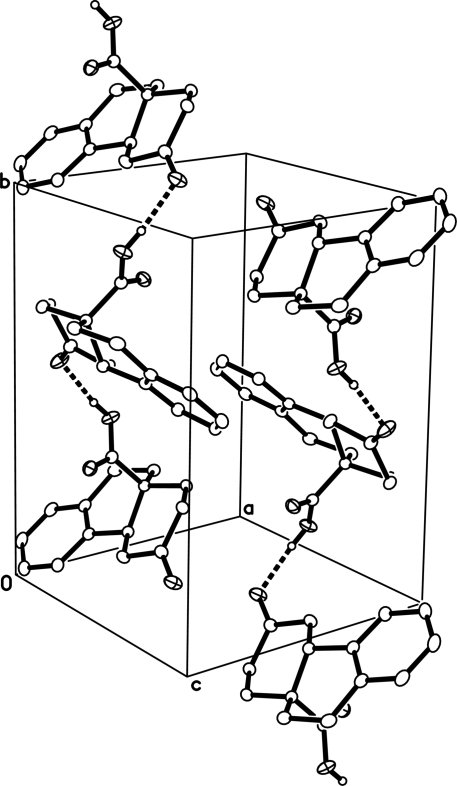
A partial packing diagram for (I), with extracellular molecules, illustrating a counter-directional pair of parallel H-bonding chains. For clarity, all C-bound H atoms are omitted. Displacement ellipsoids are drawn at the 30% probability level.

## supplementary crystallographic information

### Comment

In ketocarboxylic acids the bias toward centrosymmetric acid pairing (Leiserowitz, 1976; Gavezzotti & Filippini, 1994; Allen *et al.*, 1999) may be suppressed when molecular inflexibility diminishes the repertoire of conformational options. Keto acids with few fully rotatable bonds thus display an increased tendency to form acid-to-ketone H-bonding chains. In this context, we describe the title compound (I), which aggregates in the less common catemer mode.

Fig. 1 shows the asymmetric unit for (I) with its numbering. The sole option for full bond rotation involves the carboxyl group, which is turned so that its carbonyl lies over the ring system, with a C4A—C10A—C11—O2 torsion angle of 39.16 (17)°. Within the asymmetric unit, the dihedral angle for ketone (C2—C3—C4—O1) *versus* carboxyl (C10A—C11—O2—O3) is 86.06 (6)°. Because (I) is not dimeric, averaging of C—O bond lengths and C—C—O angles by disorder is precluded, and these values [1.2121 (17) & 1.3232 (17) Å] resemble ones typical for highly ordered carboxyls (Borthwick, 1980).

Fig. 2 illustrates the packing. The carboxyl-to-ketone H bonds proceed among molecules screw-related in b, generating two parallel single-strand helical catemers for each cell. These chains are centrosymmetrically related and thus counter-directional. For the ketone and carboxyl groups involved in each intermolecular H bond (Table 1), the C2—C3—C4—O1 *versus* C10A'-C11'-O2'-O3' [symmetry = 0.5 - *x*,-1/2 + *y*,0.5 - *z*] dihedral angle is 69.01 (6)°.

We characterize the geometry of H bonding to carbonyls using a combination of the H···O=C angle and the H···O=C—C torsion angle. These describe the approach of the H atom to the O in terms of its deviation from, respectively, C=O axiality and planarity with the carbonyl. In (I) these angles are 117° for H···O=C and -6.5° for H···O=C—C, extremely close to the "ideal" angles of 120 and 0°.

Within the 2.6 Å range we survey (Steiner, 1997), three intermolecular C—H···O=C close contacts were found in the packing, involving both the ketone and the carboxyl group. (Table 1).

Compound (I) is derived from the Δ^4^ isoskeletal unsaturated keto acid whose structure we have previously reported (Miller *et al.*, 1999), and the molecular shapes of these two compounds are so similar that (I) was found to be isomorphous with the prior material.

### Experimental

1-Tetralone was carbomethoxylated and then subjected to Robinson annulation as described by Thompson & McPherson (1977). The resulting unsaturated keto ester was hydrogenated over a Pd/C catalyst, after which Jones oxidation was employed to correct for overreduction. Mild saponification, modeled on that described by Thompson & Shah (1983), provided (I), which was sublimed and crystallized from diethyl ether to give the crystal used, m.p. 460 K. The solid-state (KBr) infrared spectrum of (I) has C=O absorptions at 1716 & 1685 cm^-1^. This peak separation is typical of the H-bonding shifts in catemers, due to, respectively, its removal from the acid C=O and its addition to the ketone. In CHCl_3_ solution, where dimers predominate, these peaks coalesce to a single absorption at 1707 cm^-1^.

### Refinement

All H atoms for (I) were found in electron density difference maps. The O—H was constrained to an idealized position with its distance fixed at 0.84 Å and *U*_iso_(H) = 1.5*U*_eq_(O). The aromatic, methylene & methine Hs were placed in geometrically idealized positions and constrained to ride on their parent C atoms with C—H distances of 0.95, 0.99 & 1.00 Å, respectively, and *U*_iso_(H) = 1.2*U*_eq_(C).

### Crystal data

**Table:**

C_15_H_16_O_3_	*F*_000_ = 520
*M**_r_* = 244.28	*D*_x_ = 1.330 Mg m^−^^3^
Monoclinic, *P*2_1_/*n*	Melting point: 460 K
Hall symbol: -P 2yn	Cu *K*α radiation λ = 1.54178 Å
*a* = 9.7172 (4) Å	Cell parameters from 4354 reflections
*b* = 12.2735 (6) Å	θ = 4.3–67.3º
*c* = 10.4867 (5) Å	µ = 0.74 mm^−^^1^
β = 102.6764 (19)º	*T* = 100 (2) K
*V* = 1220.20 (10) Å^3^	Block, colourless
*Z* = 4	0.41 × 0.38 × 0.36 mm

### Data collection

**Table:**

Bruker SMART CCD APEXII area-detector diffractometer	2106 independent reflections
Radiation source: fine-focus sealed tube	2019 reflections with *I* > 2σ(*I*)
Monochromator: graphite	*R*_int_ = 0.019
*T* = 100(2) K	θ_max_ = 67.8º
φ and ω scans	θ_min_ = 5.6º
Absorption correction: multi-scan(SADABS; Sheldrick, 2001)	*h* = −11→11
*T*_min_ = 0.750, *T*_max_ = 0.775	*k* = −14→11
7219 measured reflections	*l* = −12→12

### Refinement

**Table:**

Refinement on *F*^2^	Hydrogen site location: inferred from neighbouring sites
Least-squares matrix: full	H-atom parameters constrained
*R*[*F*^2^ > 2σ(*F*^2^)] = 0.039	*w* = 1/[σ^2^(*F*_o_^2^) + (0.061*P*)^2^ + 0.4428*P*] where *P* = (*F*_o_^2^ + 2*F*_c_^2^)/3
*wR*(*F*^2^) = 0.108	(Δ/σ)_max_ < 0.001
*S* = 1.05	Δρ_max_ = 0.25 e Å^−^^3^
2106 reflections	Δρ_min_ = −0.20 e Å^−^^3^
165 parameters	Extinction correction: SHELXTL (Sheldrick, 2008), Fc^*^=kFc[1+0.001xFc^2^λ^3^/sin(2θ)]^-1/4^
Primary atom site location: structure-invariant direct methods	Extinction coefficient: 0.0050 (8)
Secondary atom site location: difference Fourier map	

### Special details

**Table:**

Experimental. crystal mounted on cryoloop using Paratone-N
Geometry. All e.s.d.'s (except the e.s.d. in the dihedral angle between two l.s. planes) are estimated using the full covariance matrix. The cell e.s.d.'s are taken into account individually in the estimation of e.s.d.'s in distances, angles and torsion angles; correlations between e.s.d.'s in cell parameters are only used when they are defined by crystal symmetry. An approximate (isotropic) treatment of cell e.s.d.'s is used for estimating e.s.d.'s involving l.s. planes.
Refinement. Refinement of *F*^2^ against ALL reflections. The weighted *R*-factor *wR* and goodness of fit *S* are based on *F*^2^, conventional *R*-factors *R* are based on *F*, with *F* set to zero for negative *F*^2^. The threshold expression of *F*^2^ > σ(*F*^2^) is used only for calculating *R*-factors(gt) *etc*. and is not relevant to the choice of reflections for refinement. *R*-factors based on *F*^2^ are statistically about twice as large as those based on *F*, and *R*- factors based on ALL data will be even larger.

### Fractional atomic coordinates and isotropic or equivalent isotropic displacement parameters (Å^2^)

**Table:**

	*x*	*y*	*z*	*U*_iso_*/*U*_eq_	
O1	0.16954 (13)	0.53956 (9)	0.02162 (9)	0.0406 (3)	
C1	−0.01276 (13)	0.72351 (11)	0.19416 (12)	0.0252 (3)	
H1A	−0.0968	0.6757	0.1741	0.030*	
H1B	−0.0461	0.7999	0.1845	0.030*	
O2	0.29511 (10)	0.78231 (9)	0.34146 (10)	0.0350 (3)	
C2	0.07805 (14)	0.70150 (11)	0.09446 (12)	0.0265 (3)	
H2A	0.0172	0.7036	0.0054	0.032*	
H2B	0.1490	0.7603	0.1005	0.032*	
O3	0.14561 (11)	0.87586 (8)	0.43269 (10)	0.0356 (3)	
H3	0.2108	0.9222	0.4436	0.053*	
C3	0.15250 (14)	0.59394 (11)	0.11443 (13)	0.0267 (3)	
C4B	0.19751 (13)	0.56160 (11)	0.49180 (12)	0.0238 (3)	
C4	0.21490 (14)	0.55868 (12)	0.25238 (13)	0.0278 (3)	
H4A	0.3090	0.5927	0.2809	0.033*	
H4B	0.2286	0.4787	0.2534	0.033*	
C4A	0.12567 (13)	0.58788 (11)	0.35068 (12)	0.0230 (3)	
H4AA	0.0417	0.5387	0.3295	0.028*	
C5	0.31064 (14)	0.48895 (11)	0.52183 (13)	0.0268 (3)	
H5A	0.3480	0.4589	0.4531	0.032*	
C6	0.36977 (14)	0.45965 (12)	0.65045 (14)	0.0305 (3)	
H6A	0.4460	0.4095	0.6689	0.037*	
C7	0.31676 (15)	0.50411 (12)	0.75139 (13)	0.0322 (4)	
H7A	0.3559	0.4841	0.8394	0.039*	
C8A	0.14555 (14)	0.60769 (11)	0.59434 (13)	0.0255 (3)	
C8	0.20659 (15)	0.57772 (12)	0.72309 (13)	0.0297 (3)	
H8A	0.1715	0.6087	0.7927	0.036*	
C9	0.02840 (15)	0.69172 (11)	0.57102 (13)	0.0287 (3)	
H9A	0.0670	0.7615	0.6108	0.034*	
H9B	−0.0455	0.6678	0.6165	0.034*	
C10A	0.06525 (13)	0.70445 (10)	0.33777 (12)	0.0226 (3)	
C10	−0.04018 (14)	0.71177 (11)	0.42685 (13)	0.0266 (3)	
H10A	−0.0841	0.7849	0.4177	0.032*	
H10B	−0.1159	0.6574	0.3980	0.032*	
C11	0.18214 (14)	0.78966 (11)	0.37191 (12)	0.0250 (3)	

### Atomic displacement parameters (Å^2^)

**Table:**

	*U*^11^	*U*^22^	*U*^33^	*U*^12^	*U*^13^	*U*^23^
O1	0.0641 (7)	0.0364 (6)	0.0228 (5)	0.0208 (5)	0.0127 (5)	0.0039 (4)
C1	0.0259 (6)	0.0238 (7)	0.0242 (7)	0.0037 (5)	0.0017 (5)	0.0005 (5)
O2	0.0286 (5)	0.0341 (6)	0.0421 (6)	−0.0062 (4)	0.0075 (4)	−0.0009 (4)
C2	0.0313 (7)	0.0254 (7)	0.0211 (6)	0.0038 (5)	0.0021 (5)	0.0029 (5)
O3	0.0500 (6)	0.0266 (6)	0.0339 (6)	−0.0128 (4)	0.0173 (5)	−0.0087 (4)
C3	0.0303 (7)	0.0273 (7)	0.0229 (7)	0.0029 (5)	0.0063 (5)	0.0024 (5)
C4B	0.0268 (6)	0.0220 (7)	0.0219 (6)	−0.0057 (5)	0.0039 (5)	0.0016 (5)
C4	0.0330 (7)	0.0274 (7)	0.0219 (7)	0.0085 (5)	0.0037 (5)	0.0019 (5)
C4A	0.0255 (6)	0.0220 (7)	0.0207 (6)	−0.0008 (5)	0.0036 (5)	0.0008 (5)
C5	0.0271 (7)	0.0282 (8)	0.0242 (7)	−0.0036 (5)	0.0038 (5)	0.0034 (5)
C6	0.0268 (7)	0.0317 (8)	0.0301 (7)	−0.0049 (6)	−0.0002 (5)	0.0086 (6)
C7	0.0332 (7)	0.0381 (8)	0.0221 (7)	−0.0130 (6)	−0.0007 (5)	0.0071 (6)
C8A	0.0288 (7)	0.0238 (7)	0.0240 (7)	−0.0089 (5)	0.0056 (5)	0.0004 (5)
C8	0.0352 (7)	0.0316 (8)	0.0224 (7)	−0.0125 (6)	0.0068 (5)	−0.0009 (5)
C9	0.0362 (7)	0.0259 (7)	0.0267 (7)	−0.0042 (6)	0.0129 (6)	−0.0015 (5)
C10A	0.0244 (6)	0.0214 (7)	0.0214 (6)	−0.0005 (5)	0.0035 (5)	0.0000 (5)
C10	0.0268 (6)	0.0250 (7)	0.0288 (7)	−0.0013 (5)	0.0078 (5)	−0.0015 (5)
C11	0.0309 (7)	0.0243 (7)	0.0185 (6)	−0.0023 (5)	0.0025 (5)	0.0030 (5)

### Geometric parameters (Å, °)

**Table:**

O1—C3	1.2210 (17)	C4A—C10A	1.5411 (18)
C1—C2	1.5328 (18)	C4A—H4AA	1.0000
C1—C10A	1.5481 (17)	C5—C6	1.3923 (19)
C1—H1A	0.9900	C5—H5A	0.9500
C1—H1B	0.9900	C6—C7	1.387 (2)
O2—C11	1.2121 (17)	C6—H6A	0.9500
C2—C3	1.4979 (19)	C7—C8	1.382 (2)
C2—H2A	0.9900	C7—H7A	0.9500
C2—H2B	0.9900	C8A—C8	1.3996 (19)
O3—C11	1.3232 (17)	C8A—C9	1.516 (2)
O3—H3	0.8400	C8—H8A	0.9500
C3—C4	1.5043 (18)	C9—C10	1.5324 (18)
C4B—C5	1.397 (2)	C9—H9A	0.9900
C4B—C8A	1.4034 (19)	C9—H9B	0.9900
C4B—C4A	1.5258 (17)	C10A—C11	1.5276 (18)
C4—C4A	1.5280 (18)	C10A—C10	1.5331 (17)
C4—H4A	0.9900	C10—H10A	0.9900
C4—H4B	0.9900	C10—H10B	0.9900
			
C2—C1—C10A	113.87 (10)	C7—C6—C5	119.64 (13)
C2—C1—H1A	108.8	C7—C6—H6A	120.2
C10A—C1—H1A	108.8	C5—C6—H6A	120.2
C2—C1—H1B	108.8	C8—C7—C6	119.55 (12)
C10A—C1—H1B	108.8	C8—C7—H7A	120.2
H1A—C1—H1B	107.7	C6—C7—H7A	120.2
C3—C2—C1	113.12 (11)	C8—C8A—C4B	119.06 (13)
C3—C2—H2A	109.0	C8—C8A—C9	118.61 (12)
C1—C2—H2A	109.0	C4B—C8A—C9	122.31 (12)
C3—C2—H2B	109.0	C7—C8—C8A	121.55 (13)
C1—C2—H2B	109.0	C7—C8—H8A	119.2
H2A—C2—H2B	107.8	C8A—C8—H8A	119.2
C11—O3—H3	109.5	C8A—C9—C10	114.65 (11)
O1—C3—C2	121.08 (12)	C8A—C9—H9A	108.6
O1—C3—C4	120.85 (12)	C10—C9—H9A	108.6
C2—C3—C4	117.93 (11)	C8A—C9—H9B	108.6
C5—C4B—C8A	118.84 (12)	C10—C9—H9B	108.6
C5—C4B—C4A	121.57 (12)	H9A—C9—H9B	107.6
C8A—C4B—C4A	119.54 (12)	C11—C10A—C10	112.27 (10)
C3—C4—C4A	114.37 (11)	C11—C10A—C4A	111.45 (10)
C3—C4—H4A	108.7	C10—C10A—C4A	107.07 (10)
C4A—C4—H4A	108.7	C11—C10A—C1	107.74 (10)
C3—C4—H4B	108.7	C10—C10A—C1	109.50 (10)
C4A—C4—H4B	108.7	C4A—C10A—C1	108.76 (10)
H4A—C4—H4B	107.6	C9—C10—C10A	112.81 (11)
C4B—C4A—C4	113.46 (11)	C9—C10—H10A	109.0
C4B—C4A—C10A	111.46 (11)	C10A—C10—H10A	109.0
C4—C4A—C10A	114.84 (11)	C9—C10—H10B	109.0
C4B—C4A—H4AA	105.4	C10A—C10—H10B	109.0
C4—C4A—H4AA	105.4	H10A—C10—H10B	107.8
C10A—C4A—H4AA	105.4	O2—C11—O3	122.73 (12)
C6—C5—C4B	121.35 (13)	O2—C11—C10A	123.85 (12)
C6—C5—H5A	119.3	O3—C11—C10A	113.33 (11)
C4B—C5—H5A	119.3		
			
C10A—C1—C2—C3	49.48 (16)	C8—C8A—C9—C10	−174.95 (11)
C1—C2—C3—O1	143.89 (14)	C4B—C8A—C9—C10	7.00 (18)
C1—C2—C3—C4	−40.45 (16)	C4B—C4A—C10A—C11	64.36 (13)
O1—C3—C4—C4A	−146.29 (14)	C4—C4A—C10A—C11	−66.43 (14)
C2—C3—C4—C4A	38.04 (17)	C4B—C4A—C10A—C10	−58.77 (13)
C5—C4B—C4A—C4	−18.50 (18)	C4—C4A—C10A—C10	170.44 (11)
C8A—C4B—C4A—C4	164.19 (12)	C4B—C4A—C10A—C1	−177.00 (10)
C5—C4B—C4A—C10A	−150.00 (12)	C4—C4A—C10A—C1	52.20 (14)
C8A—C4B—C4A—C10A	32.69 (16)	C2—C1—C10A—C11	66.13 (14)
C3—C4—C4A—C4B	−174.25 (11)	C2—C1—C10A—C10	−171.50 (11)
C3—C4—C4A—C10A	−44.43 (16)	C2—C1—C10A—C4A	−54.81 (14)
C8A—C4B—C5—C6	1.67 (19)	C8A—C9—C10—C10A	−35.51 (16)
C4A—C4B—C5—C6	−175.66 (12)	C11—C10A—C10—C9	−61.41 (14)
C4B—C5—C6—C7	−0.7 (2)	C4A—C10A—C10—C9	61.21 (14)
C5—C6—C7—C8	−0.6 (2)	C1—C10A—C10—C9	178.96 (11)
C5—C4B—C8A—C8	−1.36 (19)	C10—C10A—C11—O2	159.27 (12)
C4A—C4B—C8A—C8	176.02 (11)	C4A—C10A—C11—O2	39.16 (17)
C5—C4B—C8A—C9	176.67 (12)	C1—C10A—C11—O2	−80.08 (15)
C4A—C4B—C8A—C9	−5.94 (18)	C10—C10A—C11—O3	−24.00 (15)
C6—C7—C8—C8A	0.8 (2)	C4A—C10A—C11—O3	−144.12 (11)
C4B—C8A—C8—C7	0.13 (19)	C1—C10A—C11—O3	96.65 (12)
C9—C8A—C8—C7	−177.98 (12)		

### Hydrogen-bond geometry (Å, °)

**Table:**

*D*—H···*A*	*D*—H	H···*A*	*D*···*A*	*D*—H···*A*
O3—H3···O1^i^	0.84	1.84	2.6675 (14)	170
C2—H2A···O2^ii^	0.99	2.45	3.3817 (16)	156
C4—H4B···O2^iii^	0.99	2.60	3.5273 (18)	156
C8—H8A···O1^iv^	0.95	2.55	3.2625 (17)	132

Symmetry codes: (i) −*x*+1/2, *y*+1/2, −*z*+1/2; (ii) *x*−1/2, −*y*+3/2, *z*−1/2; (iii) −*x*+1/2, *y*−1/2, −*z*+1/2; (iv) *x*, *y*, *z*+1.
